# Online resources for dissemination and implementation science: Meeting demand and lessons learned

**DOI:** 10.1017/cts.2018.337

**Published:** 2019-01-14

**Authors:** Bryan S. Ford, Borsika Rabin, Elaine H. Morrato, Russell E. Glasgow

**Affiliations:** 1 ACCORDS Dissemination and Implementation Science Program, School of Medicine, University of Colorado Anschutz Medical Campus, Aurora, CO, USA; 2 Department of Family Medicine and Public Health, School of Medicine, University of California San Diego, La Jolla, CA, USA; 3 Colorado School of Public Health, University of Colorado Anschutz Medical Campus, Aurora, CO, USA; 4 Department of Family Medicine, School of Medicine, University of Colorado Anschutz Medical Campus, Aurora, CO, USA

**Keywords:** Dissemination and implementation science, training, online resources, education, translational research.

## Abstract

A dramatically increased interest in dissemination and implementation (D&I) science, with relatively few training programs for D&I scientists, highlights the need for innovative ways to deliver educational materials, training, and resources. We described nine interactive, web-based D&I science resources appropriate for trainees and Clinical and Translational Science Awards. We used audience feedback and design thinking to develop resources iteratively. Primary target users are T3–T4 researchers, although T2 researchers can benefit from “designing for dissemination” resources. Workforce development resources were used in D&I science workshops, as stand-alone, self-directed resources, and for consultations and trainings. We assessed resource design (purpose, functionality), usage, user experience and engagement. Educational resources addressed included: D&I science basics, pragmatic trials, getting proposals funded, designing for dissemination, and D&I science theory selection. We reviewed the purpose, functionality, status, and usage of these interactive resources. All resources engaged users; provided interactive feedback for learners; and linked users to additional learning resources. Online resources can be valuable for preparing clinical and translational mentees for research consultations, as follow-up training activities, and as D&I workforce development resources. The resources described are publicly available and we encourage their use, further development, and evaluation by Clinical and Translational Science Awards and other programs.

## Background

Clinical translation requires dissemination and implementation (D&I) at each step of the translational pathway. Yet, translation is a well-established leaky pipeline where only 14% of evidence-based practices are implemented into real-world practice. Perhaps worse, it takes an average of 17 years to implement even this small minority of evidence-based practice [1]. D&I science breaks down barriers between research and practice and has grown dramatically as a discipline over the last two decades [2].

An explicit goal of the National Institutes of Health (NIH) Clinical and Translational Science Awards (CTSA) Program (https://ncats.nih.gov/ctsa) supports translational research and training and to disseminate successful research tools and solutions (NIH, CTSA grant PAR-18-464). However, the small number of existing D&I science training programs cannot meet the mounting demand from researchers to learn best practices [3, 4]. As a result, there is an acute need for effective, low-cost ways to provide training on research design and methods to translational investigators and trainees incorporating D&I science.

While national D&I science training programs exist (Training Institute for Dissemination and Implementation Research in Health, Implementation Research Institute, Mentored Training for Dissemination & Implementation Research in Cancer, and National Heart, Lung, and Blood Institute K12), access is limited to a small subset of applicants. There are Web sites (National Cancer Institute (NCI), University of North Carolina Implementation Science Exchange, and Society for Implementation Research Collaboration), online newsletters (Implementation Science), and still local CTSAs strive to meet the educational gap. To our knowledge, an integrated, comprehensive suite of interactive resources covering key aspects of D&I science does not yet exist. One Web site does provide a set of published resources—the Washington University CTSA program. Their Web site includes resources on several topics in D&I science including design, implementation outcomes, implementation strategies, and grant writing. A notable limitation of those resources is that they are not interactive.

Interactive web tools are a method of bi-directional communication that allow for user input and can create a tailored, personalized user learning experience. Interactivity allows users to navigate to areas of specific personal need, thus providing a more individualized way to tailor information [5]. Providing immediate, actionable feedback also increases the relevancy of the learning. As noted by Darnell *et al*. [6], the need for interactive resources is vital to facilitate uptake and application of new skills.

The purposes of this article are to: (a) describe interactive and online D&I resources that are publicly available to all CTSAs developed by our research group, (b) summarize the approaches, platforms, technologies, experiences, and lessons learned from the development and delivery of these interactive aids, and (c) discuss directions for future development and evaluation of such resources, including their use in mentoring, for those new to D&I science, and as part of research consultations.

### Overview of the Design of Our Interactive D&I Science Resources

Our Adult and Child Consortium for Health Outcomes Research and Delivery Science (ACCORDS) D&I Science Program at the University of Colorado Denver (goo.gl/vBjvnC), in partnership with the local CTSA, the Colorado Clinical and Translational Sciences Institute and other collaborators, has developed a series of interactive, online resources to address the need for accessible D&I science training. This suite of online tools provides an integrated series of resources for researchers and trainees having different levels of D&I experience. Most CTSAs do not have the number of faculty mentors or resources in D&I to meet the expanding need and escalating demand [7]. Our collection of interactive resources provides an integrated series of tools that collectively address key needs and D&I competencies [8]. Our resources address the following needs for those learning and conducting D&I research: overview and introduction to the field (Introduction to D&I Science eBook), conceptual and theoretical basis (D&I models Web site, Reach, Effectiveness, Adoption, Implementation, and Maintenance (RE-AIM) Web site), methods and measures (Pragmatic eBook, My Own Health Report Tool), tips for successful D&I grants (Tips for Getting Funded eBook), example studies, (My Own Health Report Tool and Key References on program homepage), and resources (links on homepage). Learning objectives include learning basic terms, selecting D&I science models and measures, improving health behaviors, applying D&I science frameworks to pragmatic research, and getting D&I science grants funded. Material development considered diverse levels of learners and tailored to discipline-specific translational scientists (e.g., oncology, dermatology, public health) and for applied use with practicing clinicians and patients. The primary translational stages targeted translation to practice (T3) and translation to population (T4) research, but the resources are also useful for translation to patients (T2) researchers wishing to “design for dissemination” and implementation into practice [9]. Designing for dissemination refers to a set of processes that are considered and activities that are undertaken throughout the planning, development, and evaluation of an intervention to increase its dissemination potential [2].

Common characteristics facilitate ease of use across resources. All of the web resources are relatively brief, engage users with feedback, allow for differentiated navigation, and provide a summary report. Online education principles such as motivation and diversity, guided development, which follows best practices in resource design [10]. User-centered design principles steered the development phase and created a more effective learning platform. Through formal postuse survey and in-person and virtual consultation, potential users gave feedback. As these end-users made requests for additions or deletions, iterative changes were made. Involving the learner and user-centered design in developing and tailoring curriculum are proven methods for engaging learners [10].

These online D&I science resources have been used to (a) supplement workshops and graduate courses sponsored by the Colorado Clinical and Translational Sciences Institute and our D&I science program, (b) serve as stand-alone, self-directed resources, and (c) to enhance our research consultation and training programs. Table 1 provides a summary and brief overview of each of the nine resources described below.

**Table 1:** Characteristics of ACCORDS interactive dissemination and implementation science resources

Interactive tool (URL)	Target audience	Platform and funder	Goal (and key features)	Comments and lessons learned
**The resource hub**				
ACCORDS D&I Web site (http://goo.gl/vBjvnC)	New/seasoned investigators in D&I	SharePoint ACCORDS and local university	Hub for resources, feature new resources and learning opportunities	Need to continually update, people navigate in different ways, more use of social media, collecting consultation requests helpful
**eBooks**				
Introduction to D&I for health researchers and practitioners (http://goo.gl/yVJgks)	Health service research professionals, both academic, and community	iON interactive Local university and CTSA	Introduction to D&I science, convey key terms, and methods	Videos and graphics useful, people do not use linearly, great need for basic D&I information
Pragmatic trials: a workshop handbook (http://goo.gl/ZAHHGQ)	Investigators looking to increase pragmatism in their research	iON interactive Local university and CTSA	Distinguish traditional health outcomes research from pragmatic, provide guides for use of pragmatic models, methods, and measures	Important need, diverse definitions of pragmatism, very popular site, frequent updates needed in rapidly evolving field
Getting funded in D&I (http://goo.gl/c93uu8)	New/seasoned investigators (two different levels) in D&I writing or resubmitting grants	iON interactive Local university and CTSA	Aid investigators in development and refinement of D&I grant proposals	Two levels of training, some interactivity, challenging to create, links to many static resources
**Other resources**				
D&I models Web site (http://dissemination-implementation.org)	D&I investigators and trainees	ASP.NET NCI original funder Kaiser Permanente Institute for Health Research ACCORDS D&I updates and maintains	Guide to D&I theories, models, and frameworks, provide guidance on selection, adaptation, and use	Rapid increase in # models (>150), pushing limits of resources on combining, very positive feedback, highly used for consultations, need to link to more measures, and consider additional characteristics of models
Make Research Matter (http://makeresearchmatter.org)	Researchers at all stages of D&I expertise, especially those new to the field	NCI Michigan Tailoring System	To provide planning aids for D&I projects, feature short video interviews with D&I experts on key topics	Found useful, tool could be more interactive, video interviews very popular, and need updating
**Cross-institutional collaborations**				
RE-AIM Web site (http://re-aim.org)	All researchers, clinicians and community leaders interested in applying RE-AIM (and other models) to plan and/or evaluate programs and policies for population impact	Web—multiple/changed over time Multiple funders-RWJF initially, then different university and volunteers	Aid in research planning and evaluation across the T1–T4 research continuum, several exercises, activities and resources for application of RE-AIM	Most mature Web site-challenges when hosting shifts, people love aids and calculators/quizzes, using site to promote related webinars and presentations, meeting opportunities, and list of RE-AIM references is valued
Grid enabled measures database D&I workspace-GEM (http://goo.gl/adKGFA)	D&I and other HSR researchers interested in practical measures related to key constructs	ASP.NET NCI	Provide information on characteristics of D&I measures, supplement much larger site	Crowd sourcing is tricky-needs large user base, availability of pragmatic measures is valued, needs updating
**Patient facing tool**				
My Own Health Report (http://myownhealthreport.org)	Adult primary care patients in diverse settings, includes those with no or multiple chronic illnesses, English or Spanish speaking	Web NIH and AHRQ	To assess and provide immediate feedback on health behavior, mental health, SDOH, and patient preferences	Found useful in cluster-randomized trial for goal settings and initial behavior change, flexibility and customization at multiple levels to local settings are key

ACCORDS, Adult and Child Consortium for Health Outcomes Research and Delivery Science; AHRQ, Agency for Healthcare Research and Quality; CTSA, Clinical and Translational Science Awards; D&I, dissemination and implementation; GEM, grid-enabled measures; HSR, Health Services Research; NCI, National Cancer Institute; NIH, National Institutes of Health; RE-AIM, Reach, Effectiveness, Adoption, Implementation, and Maintenance; RWJF, Robert Wood Johnson Foundation; SDOH, Social Determinants of Health; URL, Uniform Resource Locator.

## D&I Science Products and Experiences

Each tool is unique in its content, purpose, and available usage data. Below we provide detail on each resource, emphasizing the experiences and opportunities for future development. All resources are free and publicly available (goo.gl/vBjvnC). The most frequent and reliable measure available, 1-month data is presented below. These data explain total users within the time detailed. Use of these data can help analyze changes made and suggest avenues to increase the resource’s impact over time. We refer to other D&I training resources available online where appropriate on our Web site. Most of these existing resources are static rather than interactive and we hope that our experiences will prove useful to others who may wish to develop interactive D&I resources to meet the growing demand.

### ACCORDS D&I Science Program Homepage—The Resource Hub (2015) (goo.gl/vBjvnC)

**Fig. 1:**
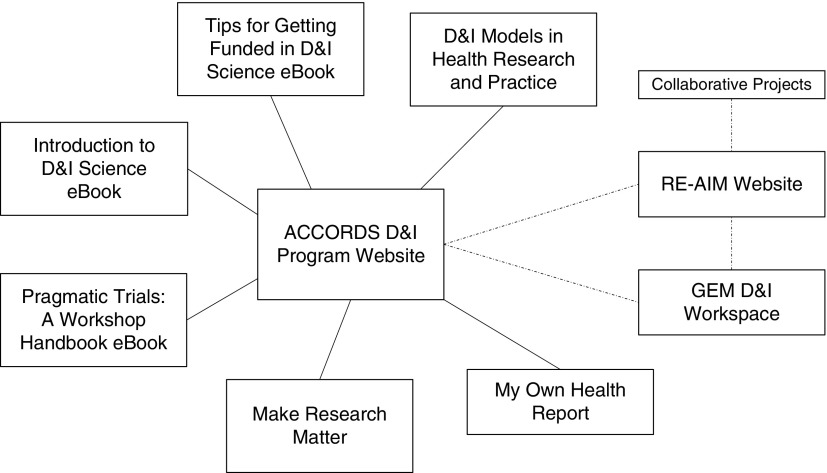
Organization of ACCORDS interactive dissemination and implementation resources. D&I, dissemination and implementation; GEM, grid-enabled measures; RE-AIM, Reach, Effectiveness, Adoption, Implementation, and Maintenance.

The University of Colorado ACCORDS D&I Science Program Web site acts as a portal to the various interactive resources. It also houses many other resources for D&I science researchers such as links to recent publications, current and upcoming learning events, training opportunities, animated learning videos, and presentations by prominent D&I science investigators. Regular, weekly updates highlight the most current work in the field. It also contains an embedded Twitter feed with the goal to increase real-time updates and to integrate social media. Fig. 1 illustrates the organization of our online resources using the D&I science program Web site as the hub.

The ACCORDS D&I Science Web site organizes our interactive aides under the “Resources” tab. We frequently communicate with local end-users to gain an understanding of how to shape each section and priority needs for new topics, information, and aids. The School of Medicine and Children’s Hospital Colorado support the Web site.

A total of 1008 total pageviews by 637 unique users in June 2018 suggests that many people come to and return to this resource multiple times. In the same month, the homepage was viewed the most with 500 views (49.6%) followed closely by the Resources page, where our interactive tools are housed, with 351 views (34.8%). Visitors from 16 different countries used the Resources page, led by the USA (271 views), Canada (46 views), and the UK (11 views).

### eBooks

We developed three online, interactive eBooks using the iON platform (https://www.ioninteractive.com/). iON is an interactive web content platform provider serving private sector clients to create data-driven product experiences without tech-savvy resources or developer code. Each book is detailed further below. Key features of all our eBooks are incorporation of multiple media methods including video segments featuring comments from leaders in D&I science, audio recordings and slides, ability of the user to see their progress through each chapter, and the option to tailor the order in which they read different sections. Most chapters conclude with a brief “key takeaway quiz” and checklist of ways to implement lessons learned, downloadable PDF documents on key issues, and links to more in-depth resources related to the information in that chapter. Original funding for development was from the Agency for Healthcare Research and Quality for a Center for Research Excellence in clinical preventive services and implementation; current funding from the National Center for Advancing Translational Sciences sustains the eBooks.

#### Introduction to D&I Science eBook (2014) (goo.gl/yVJgks)

This resource helps beginning users understand how best to disseminate, implement, and evaluate evidence-based healthcare and prevention interventions, while avoiding the implementation of inappropriate or unnecessary services. The introduction eBook is a D&I science navigation guide. It is divided into five sections or “chapters”: why D&I is important, definitions, theories and concepts, strategies and tools for designing successful D&I interventions, recommendations for evaluation design and measurement, and tips for success—for researchers and practitioners.

Specific lessons learned on this and our other eBooks are that: the iON format and programming flexibility is able to incorporate and seamlessly integrate a variety of media, but since this is a proprietary online platform not familiar to most programmers or webmasters, a learning curve exists and start-up costs are involved. Informal user feedback proved fruitful and we plan to continue using them to catalog and consolidate the learnings from future workshops. With 104 users in June 2018, users navigated to multiple segments or “chapters” 62 times. Further usage data show that the definitions pages and the key models and takeaways pages are the most popular; all with pageviews near 30.

#### Pragmatic Trials: A Workshop Handbook eBook (2015) (goo.gl/ZAHHGQ)

Pragmatic research methods and measures, while strongly related to and very frequently used by D&I researchers, extend to areas beyond D&I. We include an eBook resource on pragmatic methods here because they are so directly relevant to evaluating interventions and policies intended for dissemination. This aid covers pragmatic research focused on methods and studies conducted under real world conditions and constraints whose purpose is to inform decisions about practice [11]. It covers pragmatic models, measures, and research methods and uses the Pragmatic Explanatory Continuum Indicator Summary [12] as a framework for systematically designing and reporting pragmatic trials in three modules: study populations and setting, research design, and real-world use. It also provides links to the separate Pragmatic Explanatory Continuum Indicator Summary-2 homepage (http://www.precis-2.org), resources, and individualized printouts.

Lessons learned: a personalized printout assessing the pragmatism of a study was very popular. The video vignettes, photos, and available presentations from leading D&I science researchers sparked engagement. Naturally, this eBook will need consistent updating as the field progresses. This remains our most popular eBook with 678 visits in June 2018, though many of these users exit to the introduction to D&I eBook from the landing page. The study designs section is one of the most popular with 13 views, while other sections in this eBook had <10 visits. This may suggest the need for more attention to the introductory resource or development of more introductory aids.

#### Tips for Getting Funded in D&I Science eBook (2017) (goo.gl/c93uu8)

This eBook builds upon on our 2017 workshop as well as the work of Washington University colleagues [13, 14] and the national Training in Dissemination and Implementation Research in Health program [15]. It consists of two different options, depending upon user’s self-selected level of D&I science experience. Users who are relatively new to D&I science or at the early phase of developing a grant complete an interactive “10 Key Ingredients Checklist” [14]. Experienced D&I science investigators complete the “Advanced D&I Proposal Tips” that provides a comprehensive list of criteria to address in a research proposal [13]. Both include individualized feedback on the extent to which mentioned proposal features are included. We recommend that both the trainee or grant applicant and their mentor or co-investigator complete these resources and discuss scores.

Lessons learned: we found this tool to be very useful for mentees and other users in evaluating early versions of their proposals. It seems of particular use (a) when completed by both the applicant and by their mentor or co-investigator(s) and (b) when completed by an applicant, such as someone applying for a K award, before and/or following a consultation with one of our team members. An effort to obtain national user feedback on this resource following wide promotion proved insightful. Despite considerable work on user-centered design, an easy to complete, brief improvement survey and relatively high levels of use (386 visits from June 2017 to June 2018), very few people completed the feedback survey. We captured user characteristics, since these were required before users could access the tool. Users were from 20 different countries among them: USA (291), UK (12), and Russia (12).

### Dissemination and Implementation Models in Health Research and Practice (2015) (dissemination-implementation.org)

This interactive Web site helps researchers and practitioners select the D&I science model(s) that best fits their research question or practice problem, adapt the model to the study or practice context, fully integrate the model into the research or practice process, and find existing measures for the model constructs. These are among the most frequently asked questions and concerns from D&I science trainees and those new to the field. The Web site utilizes and expands upon the seminal review of D&I science models by Tabak *et al*. [16]. The main variables describing models include its name, primary focus on dissemination or implementation activities, construct flexibility, socioecological levels included, researcher and/or practitioner focus, number of times cited, primary reference(s), and examples of applications of the model.

This Web site currently includes close to 100 D&I science models including those from the Tabak *et al*. article, a review by Mitchell *et al*. [17], and a number of additional models. A network of D&I scientists, including some model developers, did the coding of model characteristics. We also abstracted constructs and subconstructs from each model, allowing for keyword search.

Lessons learned: this resource has been our most popular with an average of 11,820 users per month between December 2017 and June 2018 and we received very positive comments from users both locally and at national meetings. A number of recent articles and book chapters [2] recommend it and the site addresses a frequent and important need of investigators and students new to D&I science. We are currently updating the site to include additional frameworks and features including a user rating and commenting function for each model. The NCI provided partial funding for the initial development of this Web site. Because of internal funding shifts, we are also now able to host the site, which will allow us to regularly review use data and make needed adjustments.

### Make Research Matter (2013) (makeresearchmatter.org)

This Web site functions as an interactive, easy to navigate toolkit that aids designing interventions for broad application. Its target audience is program developers who want to design D&I science products, achieving wide adoption, consistent implementation and sustained use over time. Its purpose is to increase the D&I potential of effective interventions and affiliated products. While developed for those in cancer communication, the planning tool is broadly applicable and not specific to that discipline or cancer.

Make Research Matter includes three main components: planning tool, resource library, and narrative library. The narrative library is unique and contains brief video vignettes that communicate lessons learned and “how-to” knowledge in narrative form that models a solution to a particular D&I problem or opportunity. Each vignette highlights a discussion with a D&I science researcher. In addition to the 16 individual interviews, it features two D&I projects from the perspective of multiple stakeholders. Keyword searches reveal video files and text transcripts.

Lessons learned: the brief video vignettes are engaging for users and an approach we intend to integrate with other resources in the future. More information on specifically what types of persons are using this (and other resources) will help us tailor and continue to adapt these online resources.

### Cross-institutional Collaborations

Resources in this section were developed collaboratively with investigators in other settings. We acknowledge the contributions and generosity of these colleagues noted in the associated references for each resource.

#### RE-AIM Web site (2004) (re-aim.org)

The RE-AIM Web site was one of our first interactive projects. The site is an ongoing joint venture among Dr Glasgow and collaborators including Drs Paul Estabrooks, Bridget Gaglio, Samantha Harden, Borsika Rabin, David Dzelwatowski, Lisa Klesges, Marcia Ory, and others who have developed, tested and used the RE-AIM planning and evaluation framework [18]. The Web site provides background on and numerous resources related to RE-AIM including definitions, example applications, self-assessment quizzes, planning tools, and a continuously updated list of RE-AIM publications and presentations. It publicizes and promotes various D&I science related events and activities, including a monthly RE-AIM webinar.

The Web site was developed with funding from the Robert Wood Johnson Foundation and since received support from and been hosted by different academic institutions. It is currently hosted by the University of Nebraska Medical Center, overseen by Drs Paul Estabrooks, Samantha Harden, and colleagues. The Web site has changed during its history, but is increasing its guidance on practical application of RE-AIM via checklists, self-assessments, and interactive quizzes to help both researchers and community members apply the framework for planning, adapting, and evaluating D&I science projects.

An early report on the site and its use was provided by Dzewaltowski *et al.* [19]. June 2018 reporting indicated that 1668 different users started 2223 sessions with an average session lasting 3 minutes. International sessions included the USA (840), UK (153), Canada (145), and Netherlands (81). In total, 73 different countries had at least one session started. The great majority of the 2223 sessions viewed the main homepage (71%) and the second highest session count was on the “What is RE-AIM” page (17%).

Lessons learned: the RE-AIM Web site continues to be popular. Most users appear to be relatively new to or have intermediate levels of familiarity with RE-AIM based on page navigation. Prior efforts to divide the Web site into resources for researchers versus clinicians/community leaders were unsuccessful. Recently, we attempted to include more social media and “blog” features to increase Web site engagement.

#### Grid-enabled Measures Dissemination and Implementation Workspace (2012) (https://goo.gl/adKGFA)

Dr Rabin spearheaded the development of the grid-enabled measures (GEM) D&I workspace with close collaboration from Drs Glasgow, Richard Moser, and other NCI and external researchers. Funding support came from the NCI. The GEM D&I workspace uses the GEMs portal, a wiki platform that allows for user contributions and editing. The GEM portal supports behavioral scientists and other stakeholders in their research and clinical practice using validated and standardized measures based on theoretical constructs. It leverages the principles of technology-mediated social participation, such as open access, collective intelligence, and data-driven decision making, to build a knowledge base that encourages and supports collaboration [20, 21]. The community-generated content on GEM consists of constructs and their associated measures [22]. The system provides the information needed—together with qualitative data from user comments—to rate and assess each construct and measure. The creation of the GEM D&I workspace involved prepopulation with constructs and measures related to D&I science and practice. Crowd-sourcing efforts helped to add supplemental measures and have multiple stakeholders rate on pragmatism. Currently, 45 constructs and 132 measures make up the workspace.

Lessons learned: our initial GEM D&I science resource received positive reviews and included participation by a variety of stakeholders: patient advocates, clinicians, decision and policy makers, and research funders. This resource needs updates and the addition of new measures. Our initial effort at crowdsourcing was only modestly successful, and we are considering a new, more engaging, effort for this activity. A total of 2564 unique users made 3656 pageviews between May 2017 and May 2018. The measures page was the most visited with 732 (20%) views in that time; 54.9% of all hits came from mobile devices. This conforms to the current trend towards using mobile devices to access web resources over traditional desktop computers.

## Patient Facing Tool

### My Own Health Report (2013) (myownhealthreport.org)

The My Own Health Report (MOHR) aid is a patient-facing, brief, evidence-based online and interactive health risk assessment and feedback system. It includes concise, validated, patient-reported items on health risk behaviors, mental health, substance use, demographics, patient preferences, and most recently, social determinants of health [23]. This Web site is recommended to users by clinics and practitioners to support individualized goal setting and health behavior change. The platform is easily adaptable and provides a concrete example of how (online) interventions can be designed for dissemination and for other D&I researchers to use. Finally, it illustrates very concretely operationalization of some of the recommendations in other tools and D&I articles such as use of brief, pragmatic measures that are actionable and provide rapid, real time feedback to multiple stakeholders.

Results from the original study are summarized elsewhere using the RE-AIM framework [23], concluding the intervention produced high levels of reach, adoption, implementation, and effectiveness.

Lessons learned: this is our only patient-facing tool, and one of our most interactive. We have the most evaluation information on MOHR of our various resources, but it is difficult to compare to the other resources in our suite since the evaluation metrics are so different. We found that the MOHR system is broadly applicable across a wide range of primary care settings and patient populations, that patients consistently complete the assessment and goal setting activities, and that both patients and providers are satisfied with their experience. We continue to work on ways to make the system more efficient and customizable to individual settings, and are currently adding assessment items on social determinants of health.

## Cross-cutting Challenges and Lessons Learned

Funding and resource availability have presented challenges, but also some opportunities to develop and maintain these interactive resources. Volunteer time essentially supports the aids with small amounts of local funding. Without dedicated staff or programmers, it is challenging to keep tools current. As a funding period runs out for a given resource, some of the content stagnates until new funds become available. These interactive products require both D&I science content expertise and some technological skills, and personnel is not always available to maintain and make changes to the tools.

To compete with commercial products and the level of interactivity, responsiveness, and opportunity for social and peer commentary expected by users, technology platforms such as iON that are sufficiently engaging, flexible, and sophisticated can increase engagement. Finding the right staff with the appropriate blend of skills, and retaining them in an academic setting with low rates of compensation relative to industry has proven challenging. Repeated experience of turn over complicates upkeep as new employees then need to learn the different and sometimes incompatible systems on the backend, making updates and adaptations slow or not possible. This lack of continuity also made data collection and analysis challenging, as shown above in the incomplete and somewhat inconsistent user data available.

The broad applicability of these D&I science resources makes them relevant to a large audience, but this creates the need to consider tailoring for different subaudiences (e.g., researchers vs. clinicians, clinical vs. public health, beginning vs. more advanced) further complicating development. Future endeavors should focus, in the planning phase and throughout, on plans for maintenance, as frequent updating, tracking, and changes are necessary. We made efforts on three of our aids to collect feedback on utility. Unfortunately, these three cases did not provide enough respondents to analyze results. The resources were not available to provide incentives. This is a common issue across mediated interventions [24]. Considerations for user data should happen at the beginning.

Based upon our experiences, we intend to make the following changes to our current suite of online resources. (1) We will provide an engaging animation overview of all the resources available. This introduction will contain directions on how to use the resources available on the Web site and to aid those new to D&I (e.g., answer the question where do I begin). (2) We will add a section for D&I trainers, mentors and experienced researchers interested in how these resources can be integrated into training programs and needs for evaluation. (3) We are updating and expanding resources for several tools (e.g., dissemination-implementation.org). (4) We are in discussion with colleagues outside of our program and university to better share our resources and reciprocally, how to use those of other training centers, especially for distance learning. Finally, (5) we are seeking funding sources to allow us to (a) conduct more user-centered design activities and collect user feedback and (b) enhance the interactivity, and incorporate more state of the art programing and interfaces.

### Discussion

The explosion of interest in D&I science [2, 25] and user experience with this suite of interactive resources suggest an important and growing need for interactive aids and complements to D&I science training. Although not formally tested, several of these resources worked well to provide background or follow-up for research consultations, especially the D&I science models, pragmatic research guide, and RE-AIM Web site.

Iterative work by our group drove development of these online resources, piloting the aids, making relatively rapid revisions, and incorporating feedback from end users. However, the majority of the feedback we received in our pilot testing was from convenience samples and short-term on only one or two versions of these products. Although useful, more systematic, quantitative procedures, and formal user-centered design processes would enhance impact. As online learning progresses, we intend to design products with guides to further learning, tailoring the experience for different learning styles, with built-in evaluation.

We have learned the importance of engaging multiple stakeholders in tool development, and those with different D&I science interests and perspectives. These groups include researchers, educators, clinicians, and patients. Diverse perspectives do not always agree, even within a homogeneous group. We used multiple stakeholder engagement procedures and now plan to do so more systematically moving forward.

Avoiding fees and memberships for use of these tools has encouraged a diverse group of users. Overall, free availability has been positive for users who say that being able to use the tools as needed and without charge is advantageous and appreciated, especially by those in low-resource settings. Users can access all these products repeatedly, and reuse results to iteratively asses their progress. Analyses of the geographical distribution of users indicates national and international interest.

As discussed above, we experienced challenges in getting users to provide evaluative feedback to improve the tools beyond the small samples involved in initial development. We are considering required feedback to use the resource or where funding allows, incenting feedback. By gathering such additional data, more accurate tracking of users and their experience can take place and tool alteration according to audience can help improve these D&I science-learning resources.

The following conclusions and recommendations seem applicable across our D&I science resources and achievable at relatively low cost. (1) Brief presentations of information, accompanied by options for more detail and self-assessments seem to produce better engagement. (2) Given limited resources, a moderate level of tailoring and interactivity are often sufficient. The expense and added development time and costs needed to show incremental value over a basic, interactive tool like a fillable PDF or self-assessment checklist remains unconfirmed. (3) It appears important to provide immediate, actionable feedback. (4) Use of video and other narrative materials and approaches seems to promote engagement.

There are several limitations to our resources and to this report. Given the meek to nonexistent levels of funding, learning algorithms, interactive response technology, and machine learning strategies are yet to be used. To date, our evaluations of these resources consist primarily of anecdotal and limited usage data, and no comparative testing of these products to alternatives has been conducted. Given the advent and pervasiveness of social media, future versions of these aids will likely need to include greater use of features such as blogs and opportunity for public comments and cross-user interactivity.

Despite these limitations, we conclude that such D&I science resources fill an important need given the rapidly expanding demand for D&I science training. Future directions for development and testing of these and similar interactive resources includes evaluation with specific audiences (e.g., new users or beginning vs. experienced researchers). In particular, we encourage use by CTSAs to help T2 researchers and those new to D&I science to obtain knowledge about key D&I science issues and for T3–T4 researchers to get help with more advanced topics such as grant proposals and combining theoretical models. We think an important opportunity and requirement is to investigate results of such resources under different levels of support and facilitation. This is also a distinct opportunity for CTSAs to supplement the development of current and creation of new interactive D&I science resources. For example, hosting the sites or tools would permit continuity, substantive adaptations and user data tracking, allowing for analytical study of impact. Finally, an assessment could be made of the match between core D&I competencies [8] and existing D&I training and online tools directing appropriate funding to development of resources in the areas where there are the largest gaps.

Our plans, in addition to updating several of these resources, include utilizing social media, increased use of graphics, animation, and video, and eventually the use of machine learning to better customize user experience. Given the wide distribution of users, opportunity for incorporation of these interactive D&I science resources into formal courses, seminars, and online courses is apparent. All of these interactive resources are publicly available without charge, and we encourage CTSAs and others to use them and provide feedback, as well as to develop and test related tools using our experiences to guide future endeavors.

## Disclosures

The authors have no conflicts of interest to declare.
